# Subjective Visual Vertical during Caloric Stimulation in Healthy Subjects: Implications to Research and Neurorehabilitation

**DOI:** 10.1155/2015/367695

**Published:** 2015-05-26

**Authors:** Martha Funabashi, Aline I. Flores, Amanda Vicentino, Camila G. C. Barros, Octavio M. Pontes-Neto, João P. Leite, Taiza E. G. Santos-Pontelli

**Affiliations:** ^1^Department of Rehabilitation Sciences, Faculty of Rehabilitation Medicine, University of Alberta, 3-48 Corbett Hall, Edmonton, AB, Canada T6G 2G4; ^2^Department of Neuroscience and Behavior, School of Medicine at Ribeirão Preto, University of São Paulo, Avenida Dos Bandeirantes 3900, 14049-900 Ribeirão Preto, SP, Brazil; ^3^Department of Ophthalmology, Otorhinolaryngology and Head and Neck Surgery, School of Medicine at Ribeirão Preto, University of São Paulo, Avenida Dos Bandeirantes 3900, 14049-900 Ribeirão Preto, SP, Brazil

## Abstract

*Background*. The subjective visual vertical (SVV) is a perception often impaired in patients with neurologic disorders and is considered a sensitive tool to detect otolithic dysfunctions. However, it remains unclear whether the semicircular canals (SCCs) are also involved in the visual vertical perception. *Objective*. The aim of this study was to analyze the influence of horizontal SCCs on SVV by caloric stimulation in healthy subjects. *Methods*. SVV was performed before and during the ice-cold caloric stimulation (4°C, right ear) in 30 healthy subjects. *Results*. The mean SVV tilts before and during the caloric stimulation were 0.31° ± 0.39 and −0.28° ± 0.40, respectively. There was no significant difference between the mean SVV tilts before and during stimulation (*p* = 0.113). *Conclusion*. These results suggest that horizontal SCCs do not influence SVV. Therefore, investigations and rehabilitation approaches for SVV misperceptions should be focused on otolithic and cognitive strategies.

## 1. Introduction

Spatial orientation requires integration of multiple sensory inputs arising from otolith organs, semicircular canals (SCCs), somatosensory system, graviceptive system, and the visual system [1–3]. One of the assessments of spatial orientation is the subjective visual vertical (SVV).

It has been widely demonstrated that patients with vestibular disorders and encephalic lesions often present pathological tilts of SVV [4–6], which can lead to significant functional disabilities. Nevertheless, it remains unclear if these perceptions are exclusively dependent on the function of the otoliths or if they are also influenced by the SCCs function. Since the comprehension of the underlying neural processes of a disability is fundamental to develop appropriate rehabilitative strategies [7], it is important to identify what sensorial systems and receptors are involved with the visual vertical perception.

Given the above, the purpose of this study was to analyze the influence of the horizontal SCCs on the static SVV in healthy subjects. The implications of the results on neurorehabilitation and on the determination of the visual perceptions in patients with encephalic disorders are discussed.

## 2. Material and Methods

Thirty healthy subjects (7 males and 23 females; mean age 21.76 ± 2.92 years), with no evidence of vestibular dysfunction and presenting nonpathological SVV tilts, were included in the study. All subjects were further assessed to confirm the absence of vestibular dysfunction and balance disorders. The local ethical committee board approved this study and written informed consent was obtained from all subjects.

### 2.1. Equipment

To assess SVV, customized software previously developed with a visual angle of 20.14° and sensibility of 0.1° was used [8]. A neck brace was used to minimize cephalic tilts during the exam [9].

The electrooculography and caloric test were performed with NEUROGRAFF Eletromedicina, VENG digital, model VECWIN (SP/Brazil). The caloric stimulations were performed until a nystagmus-characteristic electrooculography recording was observed. With the stimulus still being provided, subjects were then instructed to open their eyes and perform SVV measurements. In order to reduce cognitive effects on the vestibuloocular reflex the subjects were instructed to do simple arithmetic calculations before and after SVV was performed when subjects had their eyes closed. The stimulus was ceased when a nystagmus-characteristic electrooculography recording was observed again, after SVV measurements. The time spent from the beginning to the end of these stimulations was recorded.

### 2.2. Procedure

Eight pilot measures of static SVV (not included in the results) were performed to account for the learning effect. The detailed procedure of SVV exam has been described elsewhere [8]. Briefly, SVV exam consisted in adjusting a virtual line composed of a row of aligned circles in the vertical position using a computer mouse. The right button turned the line into the clockwise direction, and the left button turned it into the counterclockwise direction. By convention, the real vertical was used as reference. From the real vertical, angular tilts of the virtual line were defined as positive if tilted clockwise and negative if tilted counterclockwise. Six SVV measurements were analyzed.

The caloric test was conducted according to stimulation techniques previously described [10]. The right ear was stimulated with constant airflow of 8 L/min, at 4°C, during 40 seconds [11]. The beginning of the tests occurred only if evident nystagmus was observed. Subjects were tested in supine position and the back inclined at 30°. The nystagmus' maximum velocity of slow phase (MVSP) was analyzed. The MVSP was considered abnormal if below 3°/s or above 51°/s [12].

### 2.3. Statistical Analysis

The average of the six SVV measures was used for the data analysis, which was conducted with the statistical program SPSS (Statistical Package for Social Sciences) 22.0 for Windows. In all tests, the criteria for statistical significance were two-tailed and set at* p* ≤ 0.05.

Shapiro-Wilk normality test was used to verify if data was normally distributed. For data presenting normal distribution, paired* t*-test was used for analysis. For data with nonnormal distribution, Wilcoxon signed rank test was used.

## 3. Results

**Figure 1:**
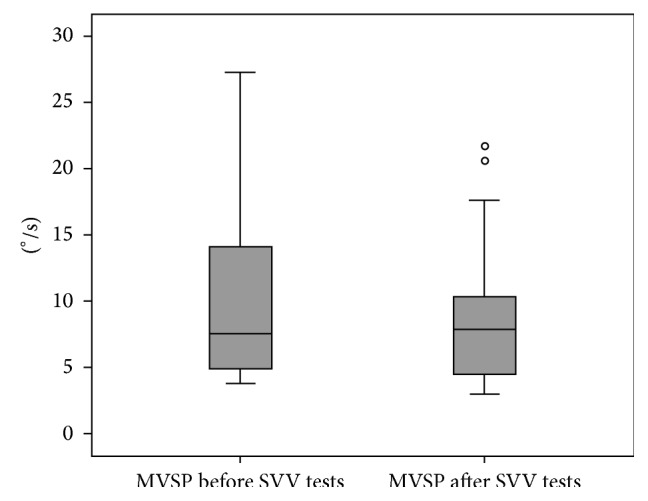
Box plot of nystagmus' maximum velocity of slow phase (MVSP) before and after subjective visual vertical (SVV) tests. Wilcoxon signed rank test:* p* = 0.067.

**Figure 2:**
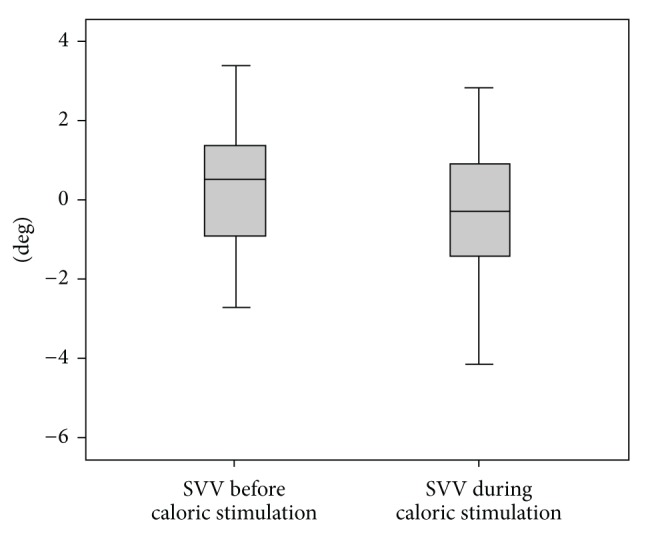
Box plot of subjective visual vertical (SVV) before and during caloric stimulation. Paired* t*-test:* p* = 0.098.

The mean MVSP before SVV measurements was 9.41 ± 6°/s (ranging from 3.8 to 27.3°/s) and after SVV measurements was 8.36 ± 5.14°/s (ranging from 3 to 21.7°/s). There was no significant difference (Wilcoxon signed rank test:* p* = 0.067) in MVSP between both situations. Furthermore, the MVSP after the end of SVV trials was above 3°/s in all subjects (Figure 1). The mean time spent to perform SVV during caloric stimulation was 1.38 ± 0.37 min. The mean SVV before and during the caloric stimulation was 0.21°  ±  1.44 and −0.41°  ±  1.54, respectively. There was no statistically significant difference between the mean SVV tilts before and during stimulation (paired* t*-test:* p* = 0.098) (Figure 2).

## 4. Discussion

This study aimed to analyze the influence of the horizontal SCCs on the static SVV in healthy subjects. By analyzing SVV during caloric stimulation, we demonstrated that the horizontal SCCs do not influence the visual perception of verticality. Similar findings have previously been reported by investigations using different methodological approaches [13, 14].

It is well known that ice-cold caloric stimulation induces endolymphatic flow in the horizontal SCC and modifies the vestibular steady-state. It has been described that the application of caloric stimulation with the head positioned at 60° of extension does not stimulate the vertical canals and it is, therefore, an evaluation of the horizontal SCCs [15, 16]. In the present study, the right ear was stimulated generating a left-beating nystagmus. This nystagmus induces vertigo and, consequently, it would be expected to result in greater SVV tilts towards the direction of the self-motion sensation in comparison to the control condition. However, there was no significant difference between SVV tilts before and during the caloric stimulation. This suggests that the afferences from the horizontal SCCs do not influence visual perception of verticality.

Given the extended caloric stimulation, excitation saturation could have potentially occurred. Indeed, during SVV tests nystagmus was not observed. However, the electrooculographic recordings after the end of SVV trials were also analyzed and the nystagmus presented mean MVSP of 7.72 ± 0.89°/s. Given that the caloric stimulus was constant throughout the entire test, the presence of a nystagmus after SVV measures indicated that the horizontal SCCs were being stimulated during all test. Therefore, the absence of nystagmus during SVV test was most likely due to ocular fixation.

The interpretation of the present findings can be useful to question whether the evaluation of the SCCs should be included in studies regarding SVV in patients with encephalic lesions. Since the horizontal SCCs do not influence SVV, the inclusion of caloric and rotatory tests in studies that evaluates SVV seems unnecessary. Nevertheless, it is important to note that isolated peripheral vestibular disorders can lead to a SVV tilt ipsilateral to the affected ear [17]. For example, in a stroke patient with lesions in the right hemisphere (that usually leads to a SVV tilt contralateral to the encephalic lesion) [18, 19] and a concomitant peripheral vestibular disorder in the left ear (that usually leads to a SVV tilt ipsilateral to the affected ear), the observed SVV tilt cannot be assumed to be caused by the encephalic lesion, to the vestibular lesion, or both. Therefore, it is important to assess neurotological function in all studies investigating visual verticality perception. However, the neurotology assessments can be restrained to otolith organs and vertical SCCs tests.

In order to elaborate new therapeutic strategies, it is essential to distinguish the underlying physiology of the targeted dysfunction [7]. Therefore, the finding of the current study that the horizontal SCCs do not influence SVV is also relevant for vestibular rehabilitation clinical practice. To date, only few studies analyzed the effects of rehabilitation in patients with SVV misperceptions [4, 19, 20]. However, none of these studies were randomized clinical trials. Some approaches that have been indicated for this purpose are orientation discrimination task on a forced-choice procedure [19], general vestibular rehabilitation programs [4], and virtual reality stimulations [20]. Since SVV misperceptions are associated with deficits of postural control, it is necessary to develop well designed and scientific based rehabilitative strategies to improve this deficit that occurs in a numerous set of patients with neurological and otoneurological disorders.
